# Evaluation of side effects of radiofrequency capacitive hyperthermia with magnetite on the blood vessel walls of tumor metastatic lesion surrounding the abdominal large vessels: an agar phantom study

**DOI:** 10.1186/2045-824X-6-15

**Published:** 2014-07-15

**Authors:** Noriyasu Kawai, Daichi Kobayashi, Takahiro Yasui, Yukihiro Umemoto, Kentaro Mizuno, Atsushi Okada, Keiichi Tozawa, Takeshi Kobayashi, Kenjiro Kohri

**Affiliations:** 1 Department of Nephro-urology, Nagoya City University Graduate School of Medical Sciences, 1 Kawasumi, Mizuho-cho, Mizuho-ku, Nagoya 467-8601, Japan; 2 College of Bioscience and Biotechnology, Chubu University, Kasugai, Japan

**Keywords:** Magnetic cationic liposome, Interstitial hyperthermia injury, Large vessels, Vascular cell damage, Agar phantom study

## Abstract

**Background:**

Magnetite used in an 8-MHz radiofrequency (RF) capacitive heating device can increase the temperature of a specific site up to 45°C. When treating a metastatic lesion around large abdominal vessels via hyperthermia with magnetite, heating-induced adverse effects on these vessels need to be considered. Therefore, this study examined hyperthermia-induced damage to blood vessel walls in vitro.

**Methods:**

A large agar phantom with a circulatory system consisting of a swine artery and vein connected to a peristaltic pump was prepared. The blood vessels were placed on the magnetite-containing agar piece. Heating was continued for 30 min at 45°C. After heating, a histological study for injury to the blood vessels was performed.

**Results:**

The inner membrane temperature did not reach 45°C due to the cooling effect of the blood flow. In the heated vessels, vascular wall collagen degenerated and smooth muscle cells were narrowed; however, no serious changes were noted in the vascular endothelial cells or vascular wall elastic fibers. The heated vessel wall was not severely damaged; this was attributed to cooling by the blood flow.

**Conclusions:**

Our findings indicate that RF capacitive heating therapy with magnetite may be used for metastatic lesions without injuring the surrounding large abdominal vessels.

## Introduction

Mammalian cell death due to fever was described by Coss et al. [1], who demonstrated that Chinese hamster ovary (CHO) cells continued to survive owing to a colony-forming capacity despite warming at increasing temperatures for a constant time. It was learned that at temperatures less than 42.5°C, the cell survival rate does not decrease rapidly, but at 43°C and above survival decreases linearly. Thus, 42.5°C is a critical temperature for cell death occurring due to fever, and hyperthermia emerged as a promising cancer therapy. As a result, various hyperthermia treatments have been attempted [2,3]. However, an unavoidable technical problem remains, i.e., even localized heating of cancer tissue at a specific temperature induces damage in the surrounding, normal tissues. In contrast, capacitive heating of tumors using a radiofrequency (RF) electric field is a method of hyperthermia that is commonly employed in actual clinical practice in Japan. However, capacitive heating is unsuitable for site-specific hyperthermia because currently available methods do not allow specific heating of the tumors alone.

In capacitive hyperthermia the normal tissue is also warmed in the part picked up with an electrode. The specific adsorption rate of electric energy depends on the electrical properties, such as permittivity and electric resistance of each tissue type. Since there are no significant differences in the electrical properties of tumor and normal tissues, it is difficult to specifically heat only the tumor. Therefore, in order to prevent excessive heating of normal tissue, “mild” heating conditions of less than 40°C. are often applied. With this method, heating of the tumor is often insufficient, and complete suppression of the tumor is rather difficult. To overcome this problem, Kobayashi et al. developed magnetite cationic liposomes (MCLs) as fine magnetic particles of submicron size for use in inductive hyperthermia [4,5]. MCLs were devised in order to improve adsorption by tumor cells and accumulation efficiency; these particles have a 10-times higher affinity for tumor cells than fine magnetic particles with no electric charge [4]. Many studies have reported the effectiveness of hyperthermia using MCLs in vivo [4,6]. We have also previously performed in vivo studies on the application of hyperthermia using MCLs for prostate cancer therapy [7,8] and have reported in vivo findings concerning its therapeutic effects on recurrent prostate cancer and bone metastases [9]. Kobayashi et al. demonstrated that magnetic nanoparticles serve as a medium that induces efficient heat generation in RF-capacitive heating [10]. In an *in vivo* experiment, they observed that heat generation using magnetic nanoparticles was similar to that using inductive heating employing an alternating magnetic field. Thus, with mild hyperthermia that is tolerable for patients, it is possible to generate heat greater than 42.5°C with MCL injection, thereby reaching the critical temperature for cancer cell death. In contrast, other tissues that have not been injected with MCL do not experience an increase in temperature beyond 42.5°C even if picked up with an electrode. Therefore, magnetic nanoparticles are considered to be a promising heat-generating medium not only for inductive heating but also for capacitive hyperthermia [11].

For lymph node metastases and local recurrence of urinary system cancers that are located deep within the trunk, tumor-specific hyperthermia using MCLs with RF-capacitive heating appears to be a possible treatment modality. However, such lesions are typically adjacent to the abdominal aorta, inferior vena cava, or the common, internal, or external iliac artery/vein. When capacitive heating is applied after the injection of MCLs into such targets, the temperature of MCL-injected lesions may reach 42.5°C as well as the surrounding tissues. If adjacent great blood vessels are injured by this heat, then massive life-threatening bleeding would occur. Therefore, before performing such RF-capacitive hyperthermia using MCLs for lymph node metastases and/or local recurrences of urothelial cancers, it is necessary to confirm that this therapy does not cause injury to the great vessels near the tumor tissue.

In this study, we prepared a model consisting of swine blood vessels and an agar phantom for simulating deep trunk lymph node treatment with capacitive heating by MCL injection, and investigated the influence of hyperthermia treatment on the blood vessels.

## Materials and methods

### Preparation of MCL

MCLs were fabricated from colloidal magnetite (a kind gift from Toda Kogyo, Hiroshima, Japan) and a lipid mixture consisting of N-(a-trimethylammonioacetyl)- didodecyl-D-glutamate chloride (Sogo Pharmaceutical Co., Ltd., Tokyo, Japan), dilauroylphosphatidylcholine, and dioleoylphosphatidylethanolamine (Sigma-Aldrich, St. Louis, MO) in a molar ratio of 1:2:2, as described previously [3].

### Swine blood vessels

Based on studies performed by research institutions following animal study guidelines, the abdominal aorta and vena cava were excised from a 3-month-old female pig being euthanized, and the cranial and caudal sides were cut to prepare vascular tubes of approximately 20 cm in length. Small branches radiating out from the arterial wall were excised, and the openings were closed by ligature/suture and application of an adhesive. Plastic connectors (Connecter 456; Tokyo Garasu Kikai Co., Ltd., Tokyo) were attached to both ends. One end was connected to a peristaltic pump (SJ-1211H; ATTO Co., Ltd., Tokyo), while the other opened into an effluent bucket, thereby preparing a liquid circuit in the blood vessel. This preparation was approved by the appropriate ethics review boards.

### Preparation of agar phantom

**Figure 1:**
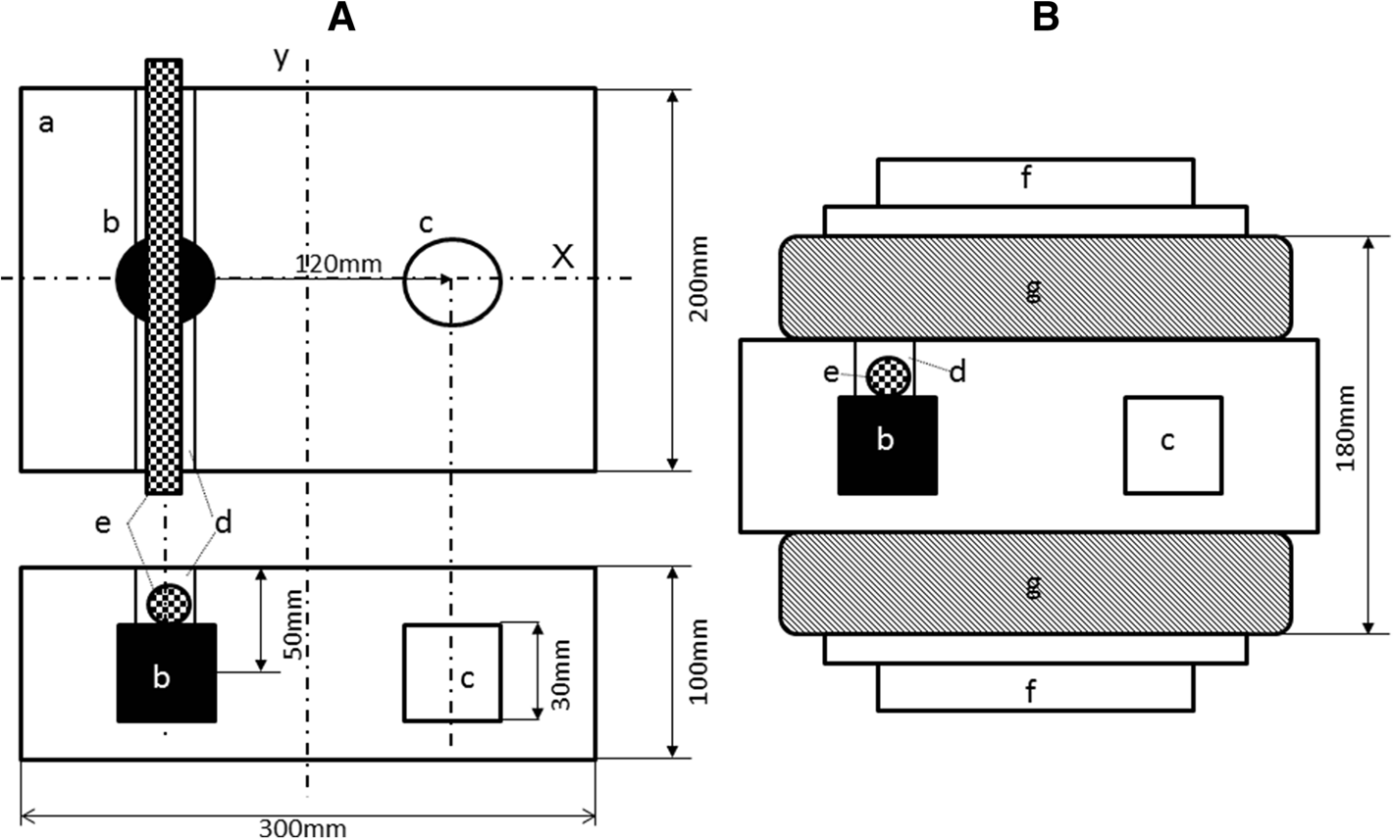
**Experimental setup.** Schemas of the agar phantom **(A)** and with the electrode setting **(B)** a: Agar phantom, b: cylindrical magnetite piece, c: control piece without magnetite, d: groove prepared for setting a blood vessel, e: vessel, f: electrode, g: water cooling pad The cylindrical magnetite and control pieces were symmetrically placed against the y-axis on the central axis of the agar phantom. A groove was made parallel to the y-axis, and a blood vessel was placed in contact with the cylindrical magnetite piece.

A 4% agar gel was used for preparation of the phantom, using agar purchased from Wako Pure Chemicals Co., Ltd., Tokyo. A small cylindrical agar piece containing the magnetite (magnetite piece) was prepared by a modification of the method described by Jordan et al [12]. In brief, the magnetite was added to liquid agar at 60°C, while stirring with a glass impeller. After mixing for 30 min, the suspension was sonicated for 15 min at 20 W using a probe-type sonicator. The sonicated mixture (20 mL) was poured into a polypropylene cylinder (28 mm in diameter) and then rapidly cooled by placing the cylinder in ice water. Figure 1A shows a schema of the phantom. Two holes were made along the centerline in the large phantom piece (300 × 200 × 100 mm), and the cylindrical magnetite piece was inserted into the piece. A groove was then made on the inserted, cylindrical magnetite piece in order to place a blood vessel in parallel with the y-axis. The blood vessel was placed in contact with the cylindrical magnetite piece. The remaining space was filled with 4% agar. An agar piece without magnetite for control experiments (control piece) was also inserted in a symmetric position with respect to the central axis of the electrode.

### In vitro RF hyperthermia

An 8-MHz RF capacitive heating device (Thermotron RF-8; Yamamoto Vinyter Co., Osaka) was used. The diameter of the pair of electrodes used for the phantom was 25 cm. The electrodes were covered with a water pad, and cooling water was circulated at 5°C. The configuration of the phantom and electrodes is shown in Figure 1B. Power control of the electrodes was performed by the standard method used in routine clinical situations by the Department of Urology, Nagoya City University Medical School.

The temperature was measured using a Teflon-coated microthermocouple that was inserted into the phantom through a 21-gauge angiocatheter. The microthermocouples were connected to the Thermotron RF-8 or a recorder (Yokogawa Electric Co., Ltd., Tokyo).

The microthermocouples were set at 2 sites: immediately beside the blood vessel located directly above the cylindrical magnetite piece, and directly above the control piece. The flow speed was set at 14 mL/s, which was the fastest rate of the peristaltic pump for both the artery and vein. Heating was continued for 30 min after the temperature directly above the cylindrical magnetite piece reached 45°C. Further, 30-min heating was applied once while saline was perfused in the artery or the vein and to another vein without saline perfusion.

After the heating was completed, the blood vessel was removed from the agar. The regions that were in contact with the cylindrical magnetite piece and adequate sections from the distant ends were excised and subjected to histological examination.

### Immunohistochemical staining and special histochemical analysis

Vascular damage was evaluated by staining elastic fibers, collagen, vascular endothelium, and smooth muscle employing Elastica van Gieson (EVG) [13], Sirius Red [14], von Willebrand factor (vWF) [15], and α-smooth muscle actin (SMA) staining [16], respectively.

Immunohistochemical staining was applied to 3 μm thick paraffin sections of the swine artery and vein. Sections were incubated for 60 min with antibodies specific to SMA (Dako Cytomation Inc.) and Factor VIII-related antigen (Dako Cytomation Inc.). Staining was achieved using the 2-step EnVision + System-HRP methodology, according to the manufacturer’s instructions (Dako Cytomation Inc.). Sections were lightly counterstained with hematoxylin to facilitate orientation. Immunostained slides were evaluated by light microscopy, and the proportion of positively stained cells (positivity) was scored from the tumor, normal tissue, and the nuclear and cell membranes.

## Results

### Heating

**Figure 2:**
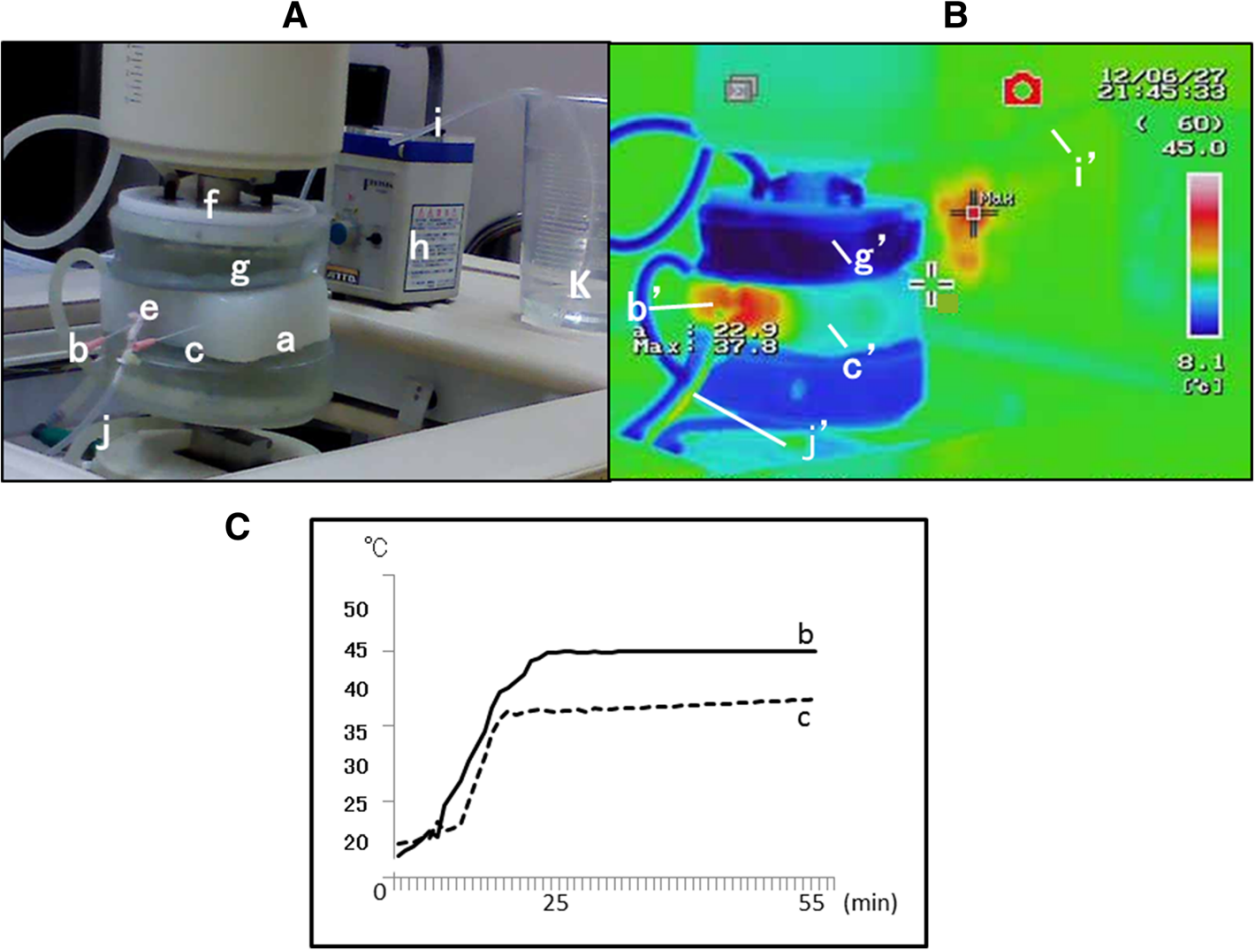
**Heating. (A)** Explanation of RF-8. a–g: same as Figure 1. h: peristaltic pump, i: silicon tube for inflow, j: silicon tube for outflow, k: saline. **(B)** Thermography. b’: Temperature display of the cylindrical magnetite piece, approximately 45°C. c’: Temperature display of the control piece, approximately 30°C. g’: Temperature display of the water cooler pad, approximately 8°C. j’: Temperature display of the silicon tube for outflow, approximately 35°C. i’: Temperature display of the silicon tube for inflow, approximately 20°C. **(C)** Time course of temperature changes in b and c. b: The temperature reached 45°C at 27 min after the initiation of heating and remained at this temperature thereafter. c: The temperature reached 35°C at 23 min and did not exceed 37°C thereafter.

Figure 2 shows the schema for heating of the agar phantom and blood vessel using RF-8. The container shown in (k) of Figure 2A contained physiological saline, and saline was pumped up through the silicon tube (i) connected to the peristaltic pump (h). Saline flowed into the blood vessel (e), passing through the agar phantom (a), and discharged into the silicon tube (j). Probes measured the temperature of the region directly adjoining the blood vessel (b) and the control piece (c). Figure 2B shows temperature measurements observed during heating using thermography (Thermography R300; NEC/Avio Co., Ltd., Tokyo). As shown by the scale on the right, red and blue colors represent 45°C and 8°C, respectively. Since 5°C cooling water was circulated in the water pad while heating, thermography displayed the color blue representing a low temperature. The color red, representing 45°C, is displayed beside the blood vessel (b’) in contact with the cylindrical magnetite piece in the agar phantom, whereas the color green, representing ~30°C, is observed for the control piece (c’). The inflow silicon tube (i) was colored blue-green (i’), showing that the inflow saline temperature was approximately 25°C. The outflow silicon tube (j), into which saline flowing out of the blood vessel passed, was colored yellow (j’), showing that the outflowing saline was heated to ~35°C. Figure 2C shows the time-course temperatures in the blood vessel (b) and control piece (c) from the point of initiation of heating using RF-8. In the blood vessel, the temperature reached 45°C at 28 min after heating initiation, while in the control piece, the temperature reached 37°C after 37 min, but no further increase in temperature was observed. Heating was continued for 30 min after the temperature reached 45°C in the blood vessel and for 30 min after it reached 37°C in the control piece.

With the power of the RF-generator turned on, the temperature of the control piece reaches 42°C. In clinical situations the control piece would represent the normal tissue packed by the electrodes of RF-8, so at 42°C. the control piece is at an intolerable temperature for the patient. Thus our hyperthermia method using MCL and Thermotron RF-8 was activated when the temperature of the magnetite-containing agar piece, which was in contact with the target organ reached 45°C., while the control piece mimicking normal tissue is kept under 37°C. This hyperthermia assembly was shown to be useful for the treatment of deeply positioned lymph node metastases.

### Change in various tissues of vascular wall

#### Elastic fibers in the vascular wall (EVG staining)

**Figure 3:**
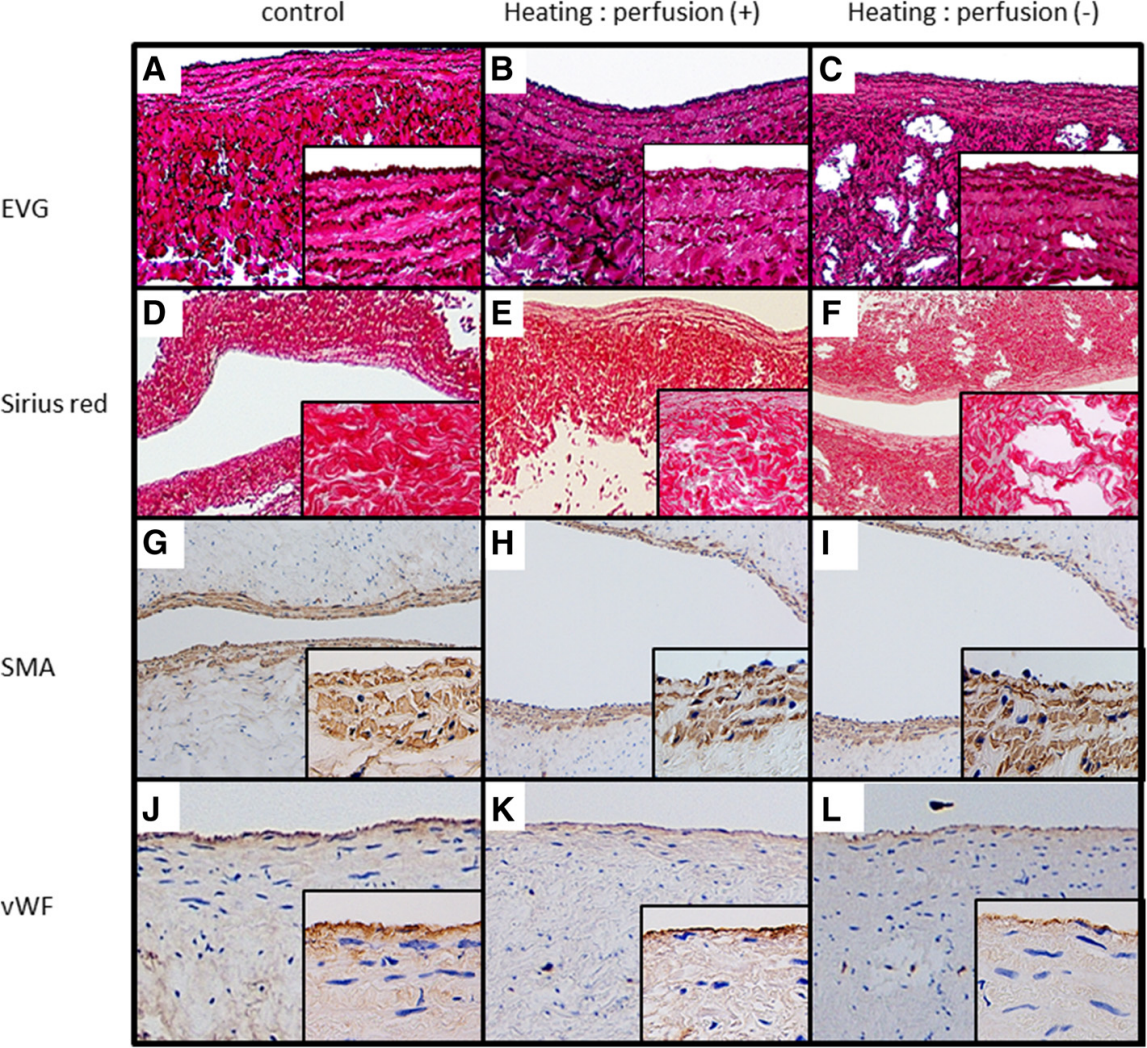
**Immunostaining and special staining of the vein vessels. (A)**–**(C)** EVG staining. Elastic fibers are stained dark purple, and collagen is stained red. **(A)** Non-heated control vein, **(B)** vein heated with perfusion, and **(C)** vein heated without perfusion. **(D)**–**(E)** Sirius red staining of the non-heated control vein, vein heated with perfusion, and vein heated without perfusion, respectively. Collagen is stained red. **(F)**–**(H)** SMA staining of the non-heated control vein, vein heated with perfusion, and vein heated without perfusion, respectively. Smooth muscle cells stained brown. **(J)**–**(L)** vWF immunostaining of the non-heated control vein, vein heated with perfusion, and vein heated without perfusion, respectively. Vascular endothelial cells are stained brown. **(A)**–**(L)**: magnification, ×100; magnification of inset photograph, ×400.

**Figure 4:**
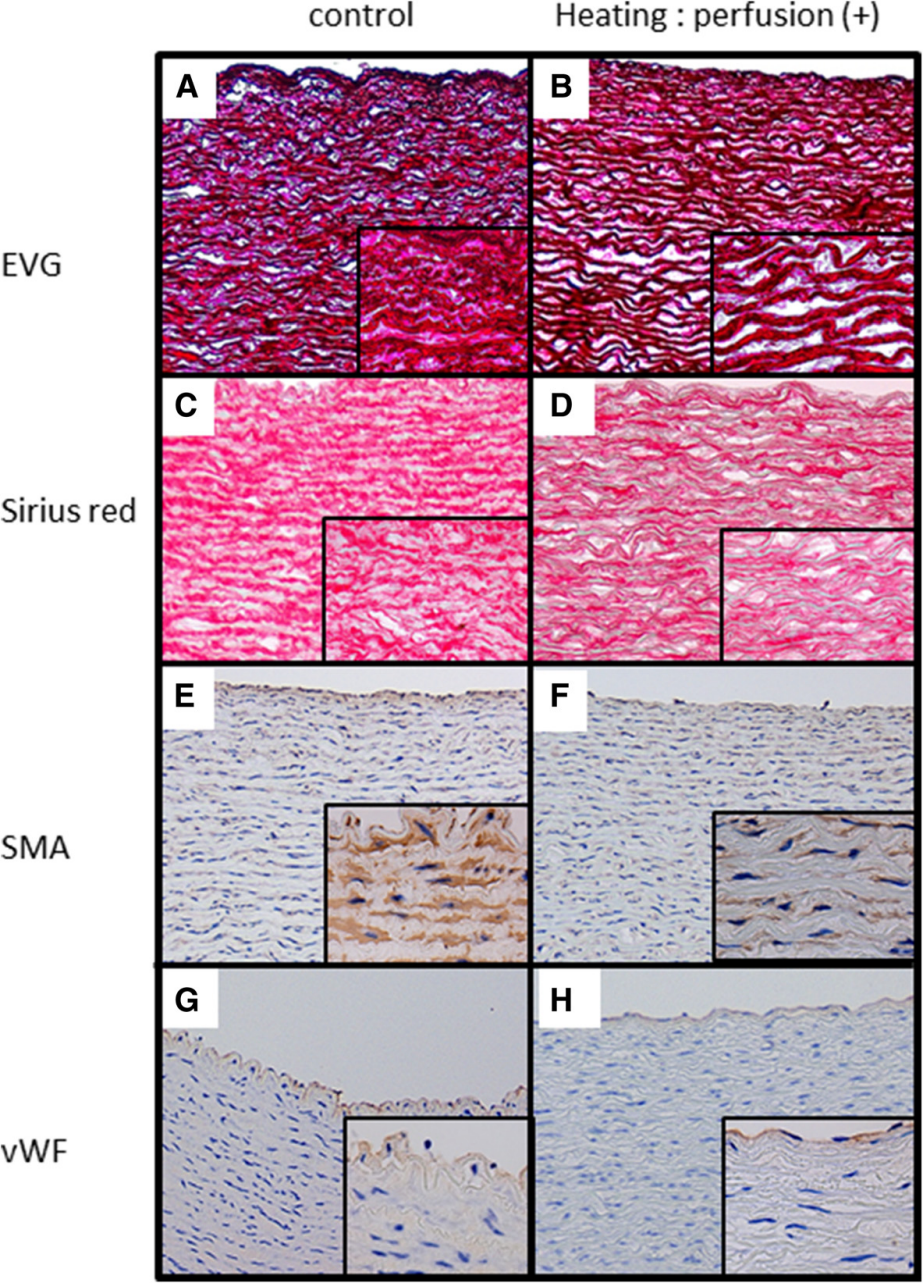
**Immunostaining and special staining of the artery vessels. (A)** and **(B)** EVG staining. Elastic fibers are stained dark purple, and collagen is stained red. **(A)** Non-heated control artery, and **(B)** artery heated with perfusion. **(C)** and **(D)** Sirius red staining of non-heated control artery and artery heated with perfusion, respectively. Collagen is stained red. **(E)** and **(F)** SMA staining of non-heated control artery and artery heated with perfusion, respectively. Smooth muscle cells are stained brown. **(G)** and **(H)** vWF immunostaining of non-heated control artery and artery heated with perfusion, respectively. Vascular endothelial cells are stained brown. **(A)**–**(H)**: magnification, ×100; magnification of inset photograph, ×400.

Elastic fibers were stained dark purple and collagen was stained red on EVG staining. In the unheated control vein, 5–6 layers of wavy, elastic fibers were noted in the tunica intima directly below the endothelium (Figure 3A). In the non-perfused, heated vein the layered structure was present, but was ruptured (Figure 3C). In the vein that was perfused with saline during heating, the layered structure of elastic fibers was not ruptured (Figure 3B). In the control arterial wall, many layers of wavy, elastic fibers and collagen-filled spaces between these elastic fibers were observed (Figure 4A). No changes were noted in the wavy, elastic fibers of the heated artery; however, the collagen filling was decreased, with empty spaces present between elastic fibers (Figure 4B).

#### Collagen in the vascular wall (Sirius red staining)

In the unheated control vein, the vascular wall was filled with collagen (Figure 3D). In the vein heated with saline perfusion, the collagen was slightly constricted, forming some empty spaces (Figure 3E). However, in the vein heated without saline perfusion, regions with complete loss of collagen were noted sporadically (Figure 3F). In the control artery, collagen filled the spaces between the elastic fibers (Figure 4C), whereas the amount of collagen was decreased in the heated artery (Figure 4D).

#### Smooth muscle of the vascular wall (SMA staining)

In the vein, 2–3 layers of smooth muscle were noted in the tunica intima. No changes were observed in the layered structure of smooth muscle in the unheated control (Figure 3G), in the vein heated with saline perfusion (Figure 3H), or in the vein heated without perfusion (Figure 3I). When smooth muscle cells were individually observed, they were narrower in the vein heated without saline perfusion than in the control. In the artery, many layers of smooth muscle were present over the entire wall. No changes were noted in the layered structure in the heated artery (Figure 4E) as compared to that in the control (Figure 4F). However, narrowing of the smooth muscle cells was observed, similar to that in the vein heated without perfusion.

#### Vascular endothelium (vWF staining)

The thickness of brown-stained, vascular endothelial cells decreased in the following order: control (Figure 3J), vein heated with perfusion (Figure 3K), and vein heated without perfusion (Figure 3L). However, no loss of endothelial cells was observed in any of the vessels. A single vascular endothelial layer was stained brown in the control artery (Figure 4G) and in the heated artery (Figure 4H), and no difference was noted in the microscopic appearance of endothelial cells.

Table 1 summarizes the results of the vascular damage that were observed. Collagen tissue in both vein and artery was damaged. However elastic fiber, smooth muscle and vascular-endothelial cells were not damaged by hyperthermia. These results indicated no serious damage of the vascular wall.

**Table 1:** Vascular damage by radiofrequency-capactive hyperthermia with magnetite

	**Vein**		**Artery**
**Saining**	**Perfusion (+)**	**Perfusion (-)**	**Perfusion (+)**
**Target tissue**			
EVG	-	-	-
Elastic fibers			
Sirius red	+	++	+
Collagen			
SMA	±	±	±
Smooth muscle			
vWF	-	±	-
Vascular-Endotherial cells			

## Discussion

The aim of the present study was to determine the effects of heating tumors located in deep regions around great blood vessels up to temperatures of 45°C by using histological and immunohistochemical examinations. Chen et al [17] previously reported a mathematical model, in which hyperthermia was simulated to coagulate tumors by inserting electrodes of an RF-ablation device, wherein heat dissemination to the surrounding region was measured, as well as determining the distance limit not affecting the adjacent great blood vessels. The influence of high heat on great vessels can be avoided in hyperthermia by using an RF-ablation device since electrodes can be freely inserted. However, the thermal effect of such devices extends only to several millimeters from the electrode, which precludes the treatment of lesions greater than 2 cm.

In our proposed hyperthermia procedure, MCLs would be injected into tumors followed by external RF irradiation. In addition to RF-induced biological heat generation, the tumor would be heated internally to temperatures up to 45°C by injecting MCLs that serve as a heating medium. These MCLs would necrotize the tumor by heating from the center, slowly expanding the region to the tumor periphery, suggesting that the temperature in the tumor periphery also rises to nearly 45°C as treatment continues [10,11]. Saline circulation was included for simulating blood flow in order to accurately evaluate injuries to the great vessels adjacent to tumors heated to 45°C.

In clinical stage, metastatic lymph nodes targeted by our proposed hyperthermia often exist around large vessels. Thus, these adjacent vessels may also be exposed to high temperatures approaching 45°C during such hyperthermic treatment of tumors. This technique differs from the hyperthermia induced by using an RF-ablation device. Therefore, the histological evaluation of vascular damage following exposure to high temperature, as performed in this study, was necessary.

Our results confirmed that the cylindrical MCL agar was heated up to 45°C using capacitive hyperthermia, as reported by Kobayashi et al. [11]. Heating to temperatures above 50°C was possible when the output of the RF generator RF-8 was increased, and the temperature in the control piece could be increased to 45°C (data not shown).

Regarding vascular wall impairment, the thin venous wall was assumed to suffer more severe damage from heat treatment than the strong arterial wall. Thus, a model of heating without saline perfusion of the vein was used, which was believed would further elevate the temperature because of the absence of a heat-releasing mechanism. Subsequently, immunohistochemical staining was performed to examine elastic fibers, collagen, and smooth muscle in the artery and veins to evaluate vascular damage. This included the vascular wall structures and vascular endothelium, which were included because they were exposed to perfusion within the vascular lumen. Elastic fibers were unchanged by heating, a finding common to the artery and the vein, while collagen was markedly degenerated following heating. Further, ruptured and defective regions were present in the vein that was not perfused with saline, in which the temperature may have risen to nearly 45°C. The number of smooth muscle cells was not reduced in either the arterial or venous wall, although individual cells became smaller.

Regarding the temperature in the vascular lumen, the saline outflow was about 30°C on thermography, suggesting that the vascular lumen, i.e., the vascular endothelial cells, were cooled to about 35°C. The lumen was also flattened in the heated artery due to elastic fiber deformity, but no changes were noted in the vascular endothelial cells. Moreover, in the vein without perfusion, no loss of endothelial cells was noted; however, the cells were narrowed. This cellular narrowing occurred to a lesser degree in the vein heated with perfusion than in the vein without perfusion.

This study used an isolated artery and vein, in which injured tissues did not recover. However, such tissues may regenerate *in vivo*, and collagen and smooth muscle may regenerate in time. Therefore, although the collagen and smooth muscle experienced some amount of degeneration, since the vascular endothelium was not ruptured and the elastic fibers were not damaged, it is conceivable that the tube structure would be maintained and fatal complications may not occur in such localized RF-capacitive hyperthermia therapy.

We submit that our hyperthermia method using MCL and Thermotron RF-8 is safe for treating deeply positioned metastatic lymph nodes.

Our present findings are expected to be useful as the preliminary data required for such an *in vivo* study.

## Conclusions

The present study indicates that RF-capacitive heating therapy with magnetite may be used for metastatic lesions that surround large abdominal vessels, without injuring these vessels.

### Ethical approval

Institutional Animal Care and Use Committee.

## Competing interests

The authors declared that they have no competing interests.

## Authors’ contributions

NK, DK, TY, KM carried out this hyperthermic experiment. NK, AO and KT carried out the histochemical examination. TK and KK advised and supported this experiment from the basic and clinical stand points. NK drafted the manuscript. All authors read and approved the final manuscript.
